# Essential Role of Linx/Islr2 in the Development of the Forebrain Anterior Commissure

**DOI:** 10.1038/s41598-018-24064-0

**Published:** 2018-05-08

**Authors:** Shaniya Abudureyimu, Naoya Asai, Atsushi Enomoto, Liang Weng, Hiroki Kobayashi, Xiaoze Wang, Chen Chen, Shinji Mii, Masahide Takahashi

**Affiliations:** Department of Pathology, Nagoya University Graduate School of Medicine, 65 Tsurumai-cho, Showa-ku, Nagoya, 466-8550 Japan

## Abstract

Linx is a member of the leucine-rich repeat and immunoglobulin family of membrane proteins which has critical roles in the development of the peripheral nervous system and forebrain connectivity. A previous study showed that Linx is expressed in projection neurons in the cortex and in cells that comprise the passage to the prethalamus that form the internal capsule, indicating the involvement of Linx in axon guidance and cell-cell communication. In this study, we found that Linx-deficient mice develop severe hydrocephalus and die perinatally by unknown mechanisms. Importantly, mice heterozygous for the *linx* gene exhibited defects in the development of the anterior commissure in addition to hydrocephalus, indicating haploinsufficiency of the *linx* gene in forebrain development. In N1E-115 neuroblastoma cells and primary cultured hippocampal neurons, Linx depletion led to impaired neurite extension and an increase in cell body size. Consistent with this, but of unknown significance, we found that Linx interacts with and upregulates the activity of Rho-kinase, a modulator of many cellular processes including cytoskeletal organization. These data suggest a role for Linx in the regulation of complex forebrain connectivity, and future identification of its extracellular ligand(s) will help clarify this function.

## Introduction

The complex connectivity between neurons in the central and peripheral nervous systems is tightly regulated by sophisticated cell-cell interactions and signaling cascades that construct neuronal circuits and transmit neuronal activity^1,2^. Previous studies have identified a number of guidance cue molecules that either attract or repulse growing axons, such as Netrin and Ephrin proteins that signal through their cognate receptors, and intercellular adhesion molecules, such as N-cadherin, NCAM (neural cell adhesion molecule), and L1, which contribute to the development of the nervous system^3^. Of these proteins, the members of the leucine-rich repeat (LRR) and immunoglobulin (LIG; also referred to as LRRIG) family of transmembrane or glycosyl-phosphatidyl inositol (GPI)-anchored proteins, which constitute a subfamily of the leucine-rich domain-containing protein family, are intriguing in that they are preferentially expressed in the central and peripheral nervous systems^4–6^. Their primary structures are comprised of various numbers of extracellular LRRs and one to three immunoglobulin (Ig) domains, both of which are known to be involved in ligand interactions and protein-protein interactions^6^. At present, the identification of ligands or binding partners for the LIG family members has been limited except for some members that bind Netrin-G, Nogo-66, and receptor tyrosine kinases (RTKs)^4,7–11^.

**Figure 1:**
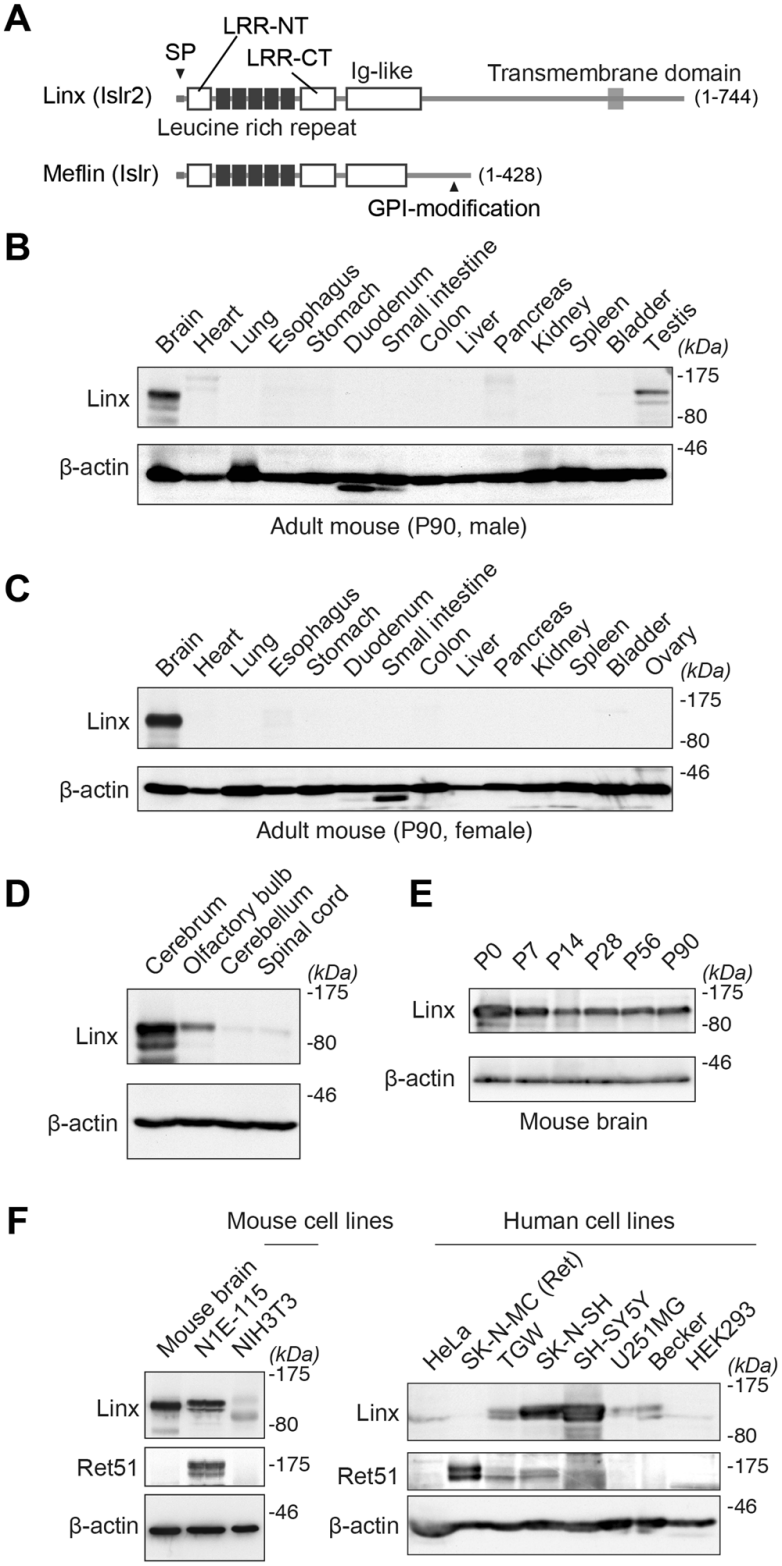
Linx expression in the forebrain and neuroblastoma cell lines. **(A)** Domain structures of Linx/Islr2 and its paralogue Meflin/Islr. Amino acid numbers are shown in parentheses. SP, signal peptide; LRR, leucine-rich repeat; LRR-NT and CT, LRR N-terminal and C-terminal cysteine-rich domains; GPI, glycosylphosphatidylinositol. **(B**,**C)** Tissue distribution of Linx in adult male **(B)** and female **(C)** mice. Lysates prepared from the indicated tissues were analyzed by Western blot with the indicated antibodies. *kDa*, kilodaltons; P, postnatal day. Full blot images are shown in Supplementary Figure S3. **(D)** Linx is preferentially expressed in the forebrain but not the hindbrain nor the spinal cord. Full blot images are shown in Supplementary Figure S3. **(E)** Linx is expressed in the brain throughout postnatal and adult stages in mice. Full blot images are shown in Supplementary Figure S3. **(F)** Linx expression in mouse and human cell lines. Lysates prepared from each mouse (left) and human (right) cell line were subjected to Western blot analysis, which revealed high levels of Linx expression in the neuroblastoma cell lines including N1E-115, SK-N-SH, and SH-SY5Y. Full blot images are shown in Supplementary Figure S3.

Linx, also termed Immunoglobulin Superfamily Containing Leucine-rich Repeat 2 (Islr2), is a type I transmembrane protein and a member of the LIG family of proteins with five tandem LRRs, an Ig-like domain, a transmembrane domain, and an intracellular domain, and that is specifically expressed in neural tissues (Fig. 1A)^4,5^. It has been reported that Linx binds to RTKs including TrkA and Ret, receptors for nerve growth factor (NGF) and glial-derived neurotrophic factor (GDNF), respectively, to modulate the intensity of their signaling cascades, although their precise binding selectivity and modes of interaction have yet to be fully elucidated^4^. Linx-deficient mice exhibit defects in axonal projections from peripheral somatosensory and motor neurons, partially mimicking the phenotypes of NGF-, TrkA- and Ret-deficient mice^4^. In addition, defects in axonal intermingling between thalamocortical and corticofugal neurons and the formation of the internal capsule (IC) were observed in the forebrain of Linx-deficient mice^12^. However, this phenotype was not fully explained by the impaired action of NGF and GDNF, suggesting that Linx interacts with other unknown ligand(s) to exert its biological functions.

We have long studied the function of Ret, which mediates GDNF signaling to regulate the development of the nervous system, kidney and gastrointestinal tract and that underlies the etiology of many human diseases^13–17^. Given the interaction of Linx with Ret and many previous studies showing that Lrig1–3 (leucine-rich repeats and immunoglobulin-like domains 1–3), other members of the LIG family, interact with epidermal growth factor receptor (EGFR) to regulate embryonic development and cancer progression^4,18,19^, we initially hypothesized that Linx could be a critical regulator of Ret function. In the present study, we first sought to characterize the expression profile of Linx in the body. We also generated Linx-deficient mice using a different approach than in a previous study, and found that they died soon after birth (P0, postnatal day 0), which was consistent with the previous report^12^. We unexpectedly found that mice heterozygous for the *linx* gene develop hydrocephalus and exhibit severe defects in the development of the anterior commissure (AC), indicating a haploinsufficiency effect of Linx during the development of the forebrain along with its function in axon path finding. Unfortunately, we could not identify any effects of Linx on the RTK signaling pathway. Instead, we identified Rho-kinase as an interacting protein with Linx, further suggesting the involvement of Linx in cytoskeletal organization and other various cellular processes during brain development.

## Results

### Tissue distribution of Linx and its expression in neuroblastoma cell lines

In a previous study that comprehensively examined the expression and distribution of the LIG family of proteins in mouse embryos by *in situ* hybridization (ISH), Linx/Islr2 was shown to be exclusively expressed in the nervous system^20^. In the present study, we first examined the tissue distribution of Linx at the protein level in adult male and female mice (Fig. 1B,C). As expected, Linx expression was specifically found in the brain with an exception in the testes of male mice. Linx expression was abundant in the cerebrum and the olfactory bulb (OB) but not in the cerebellum nor spinal cord, indicating a role for Linx in the function or development of the forebrain (Fig. 1D). Linx is expressed throughout postnatal and adult stages, suggesting multifaceted roles in development as well as homeostasis, neurogenesis, or physiological functions of the nervous system (Fig. 1E). Linx was also detected in several mouse and human neuroblastoma cell lines (N1E-115, TGW, SK-N-SH, and SH-SY5Y) but not in non-neural cell lines including NIH3T3 (fibroblasts), HeLa (cervical cancer cells), and HEK293 (embryonic kidney epithelial cells) (Fig. 1F). Although marginal, Linx expression was faintly detected in human glioma cell lines including U251MG and Becker. Combined, these data along with a previous ISH study, led to the hypothesis that Linx functions primarily in neuronal but not glial cells during both developmental and adult stages.

### Development of hydrocephalus in Linx-deficient mice

**Figure 2:**
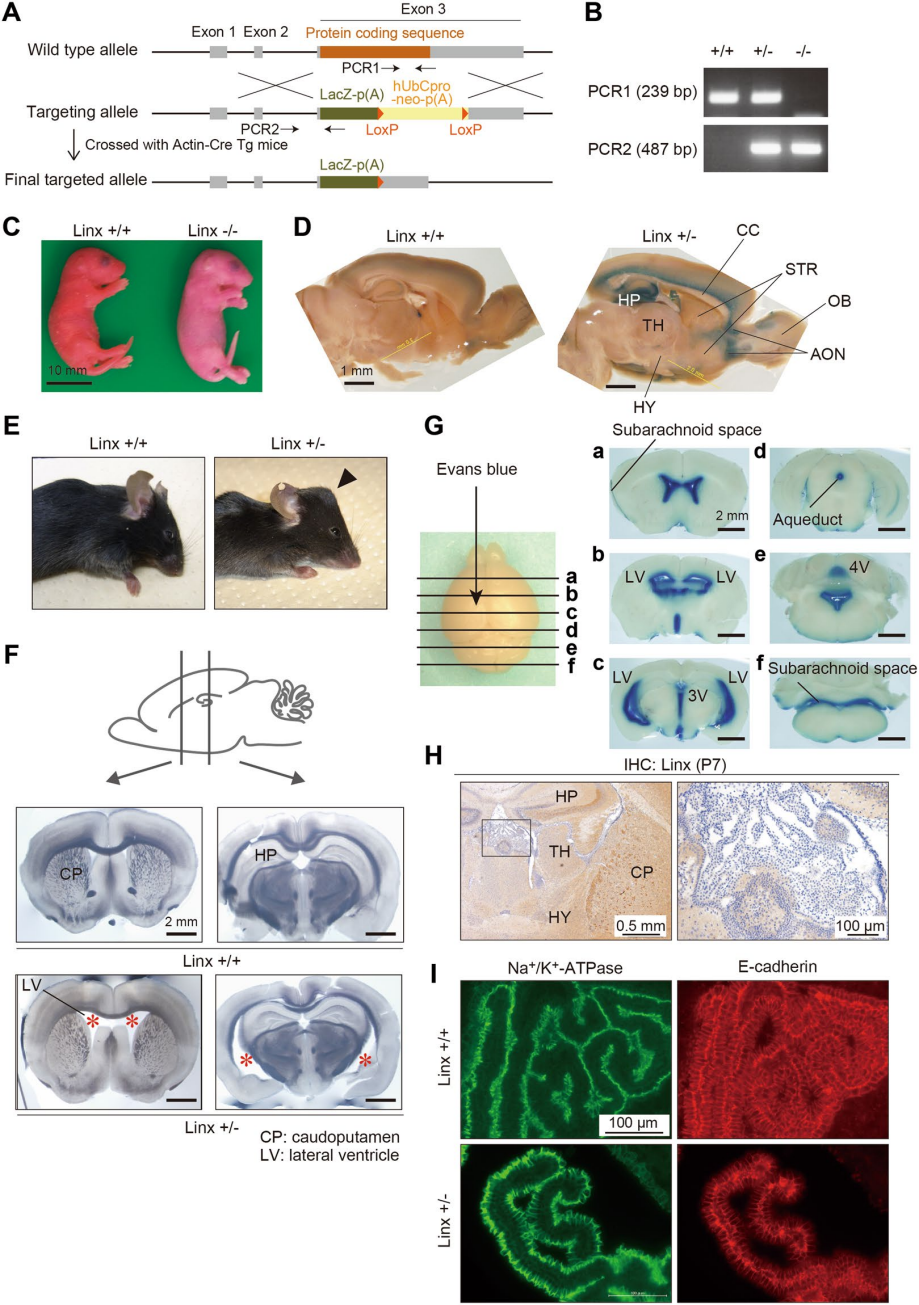
Linx^+/−^ mice exhibit severe hydrocephalus. **(A)** A schematic illustration showing the strategy for targeting the *linx* (*islr2*) gene in mice, as designed by the KOMP. Note that exon 3 of the *linx* gene encodes an entire ORF. **(B)** Representative data from genotypic PCR shows the complete deletion of the wild-type alleles in Linx^−/−^ mice. **(C)** Linx^−/−^ mice die perinatally of unknown causes and have dry skin. A representative view of newborn mice just after birth is shown. **(D)** X-gal staining of P26 brain slices prepared from Linx^+/−^ mice shows the expression of Linx in a region near the apical surface of the cortex, hippocampus (HP), and the AON. STR, striatum; HY, hypothalamus; CC, corpus callosum. **(E)** Gross appearance of the heads of P14 wild-type and Linx^+/−^ mice. Note the visible skull bump (arrowhead) found in Linx^+/−^ mice. **(F)** Transmitted light images of coronal sections of P21 mouse brains show an enlarged LV (asterisks) in Linx^+/−^ (lower panel) but not wild-type (upper panel) mice. CP, caudoputamen. **(G)** The distribution of Evans dye injected into the LV of the Linx^+/−^ P21 mouse brain, showing that Linx^+/−^ mice develop a communicating form of hydrocephalus. **(H)** No apparent expression of Linx in the ependymal cells of the choroid plexus. A boxed area was magnified in the adjacent panel. **(I)** No apparent differences in the expression and localization of Na^+^/K^+^-ATPase (left) and E-cadherin (right), markers for the apical and lateral membranes, respectively, in the ependymal cells of the choroid plexus between Linx^+/+^ (upper panel) and Linx^+/−^ (lower panel) mice.

Previous studies showed that Linx-deficient mice exhibit defects in the formation of the IC and axonal extensions of the peripheral nervous system^4,12^. We also generated Linx-deficient mice using correctly targeted embryonic stem (ES) cells available from the KOMP (Knock Out Mouse Project) repository, which enabled us to monitor the expression of Linx by X-galactosidase (X-gal) staining in both homozygous and heterozygous mice (Fig. 2A,B). We found that Linx-deficient (Linx^−/−^) mice die during the late embryonic stage or soon after birth at P0 (Fig. 2C), which is consistent with a previous report^4^. X-gal staining of postnatal brain tissue from P21 heterozygous (Linx^+/−^) mice identified the expression of Linx in the dentate gyrus and the CA3 and CA1 regions of the hippocampus and in the region near the apical surface of the cortex, suggesting the involvement of Linx in neocortical projection and hippocampal function (Fig. 2D). Linx expression was also detected in a region of the brain rostral to the striatum (STR), which appears to correspond to the anterior olfactory nucleus (AON), further suggesting a role for Linx in the development or function of the olfactory cortical circuits (Fig. 2D).

One of the phenotypes we observed in the Linx^−/−^ mice but that was not described in the previous study was the development of severe hydrocephalus, which could be a contributor to the embryonic or perinatal death of these mice (data not shown)^12^. Of note, Linx^+/−^ mice also developed hydrocephalus postnatally with a 100% penetrance (Linx^+/−^ mice, 50/50; wild-type [Linx^+/+^] mice, 0/50), indicating the haploinsufficiency of the *linx* gene for brain development and function (Fig. 2E). Hydrocephalus and the enlargement of the lateral ventricle (LV) were confirmed by observations of coronal sections through the brain of Linx^+/−^ mice using transmitted light microscopy (Fig. 2F). Injection of Evans dye into the LV to visualize the circulation of cerebral spinal flow (CSF) *in vivo* did not show any impediment in either intra- or extra-ventricular CSF flow (Fig. 2G). This result thus excludes the possibility that Linx^+/−^ mice develop a non-communicating form of hydrocephalus. Further histological analyses showed that Linx is not expressed in the ependymal cells of the choroid plexus, and the expression and localization of Na^+^/K^+^-adenosine triphosphatase (Na^+^/K^+^-ATPase) and E-cadherin, markers for the apical and lateral membranes of those cells, respectively, were not affected in Linx^+/−^ cells, suggesting that Linx is not involved in the function of the ependymal cells (Fig. 2H,I).

### Defective development of the AC in Linx^+/−^ mice

**Figure 3:**
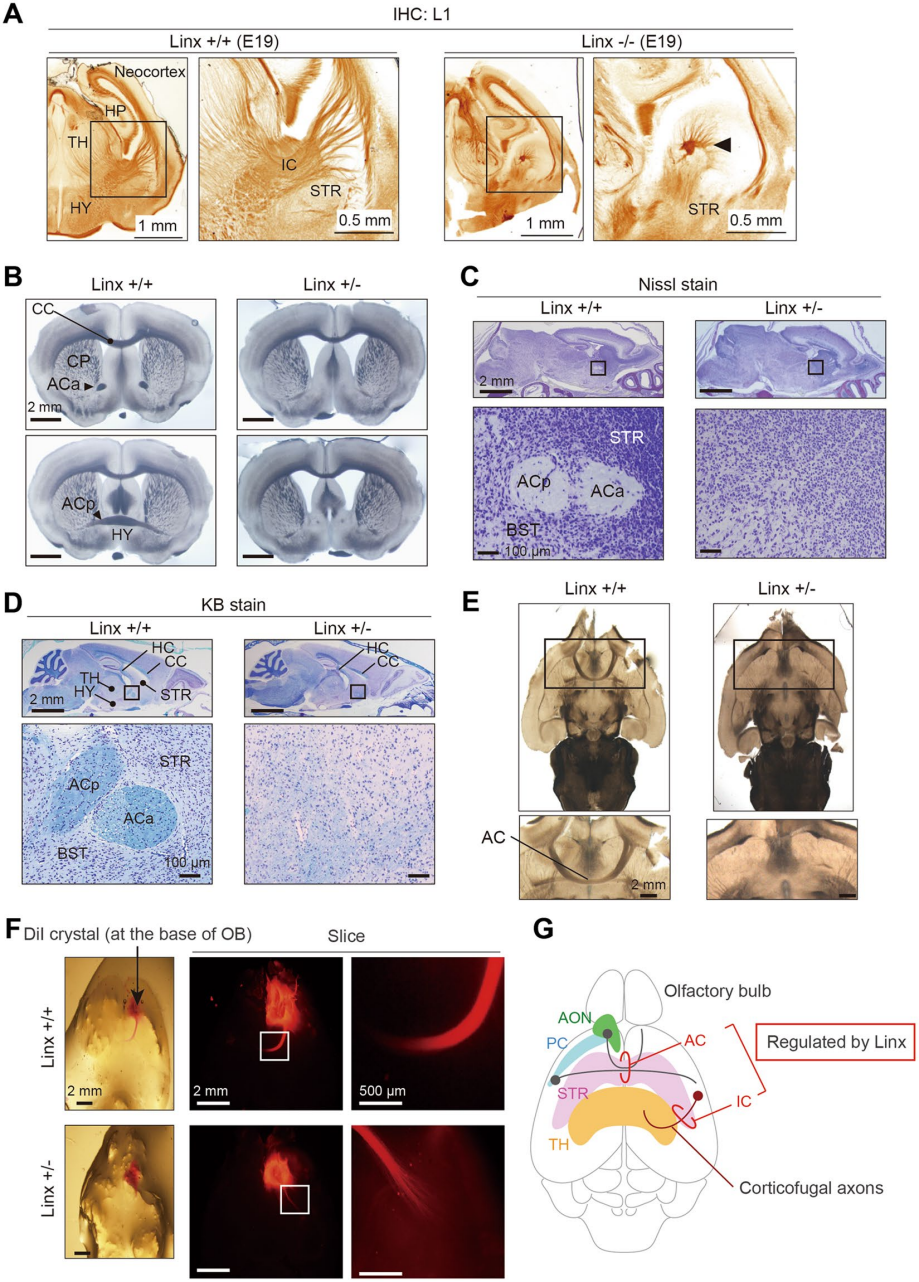
Defective AC development in Linx^+/−^ mice. **(A)** A defect in IC development found in Linx^−/−^ mice. The coronal sections of brains from Linx^+/+^ (left) and Linx^−/−^ (right) E19 embryonic mice were stained for L1, a neuronal adhesion molecule, to visualize the IC. An arrowhead denotes the remnants of the descending component of the IC. Boxed areas were magnified in the adjacent panels. **(B–D)** Transmitted light **(B)**, Nissl-stained **(C)**, and KB-stained **(D)** images of coronal sections from P30 mouse brains showing the defects in the AC of Linx^+/−^ mice. ACp, posterior branch of the AC; ACa, anterior branch of the AC; TH, thalamus; HC, hippocampal commissure; BST, bed nuclei of the stria terminalis. **(E)** Horizontal sections from the brains of wild-type (left) and Linx^+/−^ (right) mice showing defective AC development in Linx^+/−^ mice. Boxed areas are magnified in the lower panels. **(F)** The axon tract from the AON was labeled with DiI crystals placed around the AON regions prepared from wild-type and Linx^+/−^ mice. The axon tract that constitutes the anterior branch of the AC was short and disrupted in Linx^+/−^ mice compared with wild-type mice. Boxed areas are magnified in the adjacent panels. **(G)** A schematic illustration representing the neural projections regulated by Linx; this is based on data from both previous^12^ and present studies.

The expression of Linx in the neocortex and region surrounding the AON (Fig. 2D) prompted speculation that Linx may be involved in the formation of neocortical circuitry or the interconnected networks of neurons in the forebrain. As previously reported^12^, we found that Linx^−/−^ mice displayed a severe defect in the development of the IC, where the thalamocortical projections that form the ascending component of the IC are aberrant (Fig. 3A). In addition, observations of serial coronal sections from the brains of Linx^+/+^ and Linx^+/−^ mice using transmitted light microscopy showed that the formation of the AC, which connects olfactory structures and the anterior and posterior piriform cortices^21^, was completely defective in Linx^+/−^ mice (Fig. 3B). Both anterior and posterior branches of the AC (ACa and ACp, respectively), which comprise axons from the AON and the anterior piriform cortex and those from the posterior piriform cortex and the amygdala, respectively, were defective in Linx^+/−^ mice. In contrast, there was no significant difference in the thickness of the corpus callosum (CC); the CC in Linx^+/−^ mice was the same size as in age-matched wild-type mice (Fig. 3B). These observations were further confirmed by Nissl and Klüver-Barrera (KB) staining, which identified complete loss of myelinated axon tracts in the regions corresponding to both the ACa and ACp (Fig. 3C,D). The defect in AC formation was also evident in horizontal section from Linx^+/−^ brains (Fig. 3E). This was further confirmed by the implantation of DiI (1,1′-dioctadecyl-3,3,3c3′-tetramethylindocar-bocyanine perchlorate) at the position of the AON in the brain of Linx^+/−^ mice, which failed to cross the midline along the AC (Fig. 3F). In addition, our preliminary observations of central sagittal section from the brain of Linx^−/−^ mice identified defective development of the hippocampal commissure and hippocampal fornix projection (data not shown). Thus, together with the previous study that showed the importance of Linx in the development of the IC, our study suggests that Linx is essential for commissural and longitudinal projections of specific neurons in the forebrain (Fig. 3G)^12^.

### Linx regulates neurite extension and cell body size

**Figure 4:**
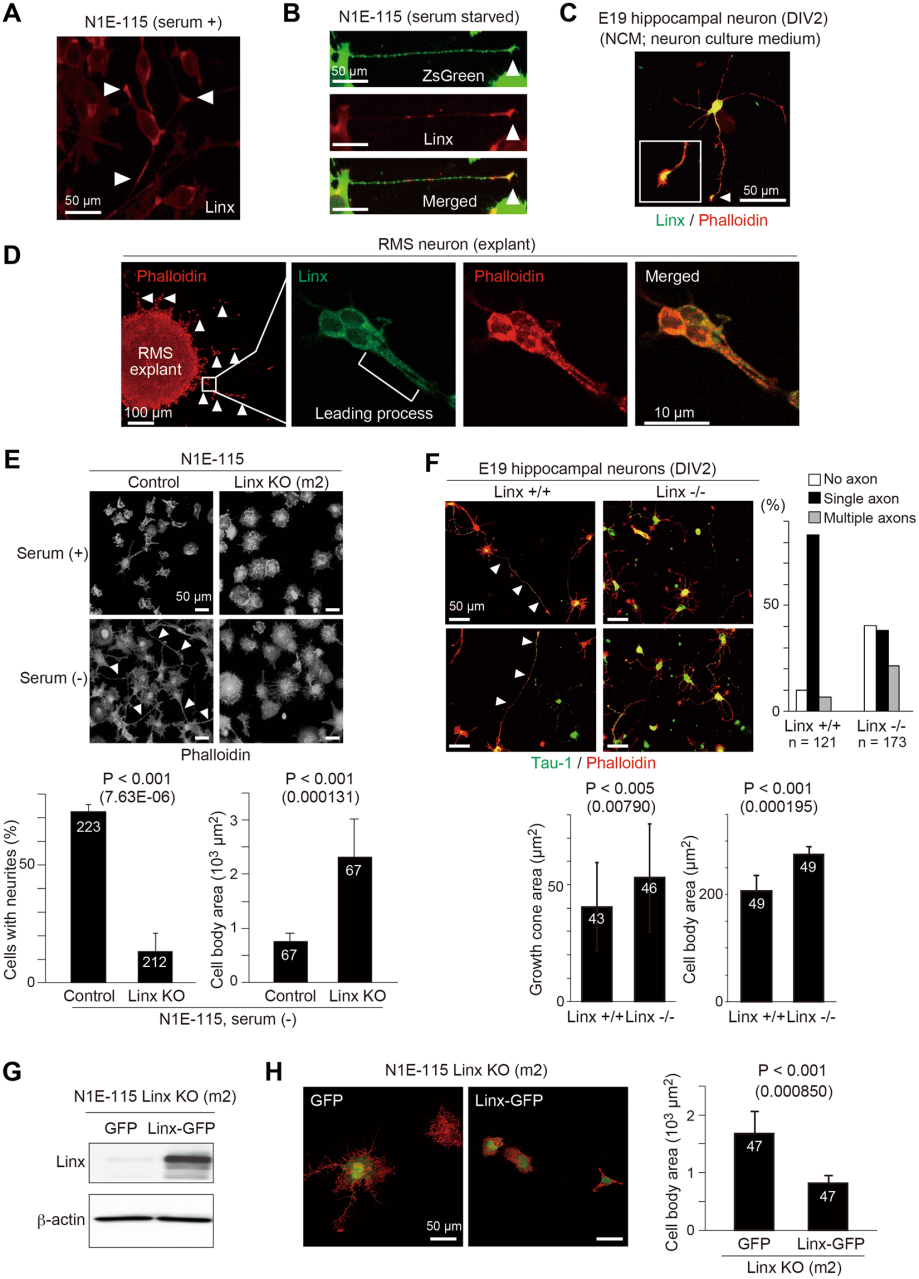
Linx regulates neurite extension and cell body size. **(A**,**B)** Linx is localized to the tips (arrowheads) of neurites in both undifferentiated **(A)** and differentiated **(B)** N1E-115 cells. In **(B)**, ZsGreen was transfected as a fill to visualize cell bodies and neurites. **(C)** Linx preferentially localizes to the growth cones (arrowhead) of growing axons in cultured primary hippocampal neurons. DIV, days *in vitro*. The growth cone is shown enlarged in the inset. **(D)** Linx localization in the leading processes and cell bodies of neuroblasts (arrowheads) that are migrating out from RMS explants embedded in Matrigel. Boxed areas are enlarged in adjacent panels. **(E)** Linx depletion leads to defective elongation of neurites in N1E-115 cells. Arrowheads indicate neurites. The graphs show the number of cells possessing neurites greater than 50 µm in length (lower left) and cell body size (lower right). Note that the average cell body size of Linx-depleted cells was much larger than control cells. The numbers in bars indicate the numbers of samples analyzed. **(F)** Hippocampal neurons isolated from the brain of Linx^−/−^ embryos at embryonic day (E) 19 show defective elongation of axons compared with wild-type (Linx^+/+^) embryos. Arrowheads indicate Tau-1-positive axons. The graphs show the number of axons identified by Tau-1 staining (upper right), growth cone area (lower left) and cell body size (lower right) of the neurons. The numbers in bars indicate the numbers of samples analyzed. **(G)** Re-expression of Linx-GFP in Linx-depleted N1E-115 cells as confirmed by Western blot analysis with an anti-Linx antibody. Full blot images are shown in Supplementary Figure S3. **(H)** Rescue of cell body size by the expression of Linx-GFP, but not GFP only, in N1E-115 cells depleted of endogenous Linx. The graph shows the quantified cell body size measured as surface area.

Previous studies have shown complicated mechanisms for the formation of the IC and AC^2,21,22^. Guidance cue molecules and cell-intrinsic machinery that drive axonal path finding are not sufficient to confer full development of the nervous system, as glial and corridor cells that comprise the tunnel or path for axonal tracts also have essential roles^2,21,22^. To gain insight into Linx function, we examined the subcellular localization of endogenous Linx by immunofluorescence in cultured N1E-115 neuroblastoma cells. The specificity of Linx antibody was confirmed by immunostaining on control N1E-115 cells and those depleted of Linx by using CRISPR/Cas9-based genome editing^23^ (Supplementary Figure S1A,B). For the depletion of Linx, we designed two guide RNA constructs (m2 and m3), and found that m2 effectively depleted Linx after antibiotic selection, which we utilized for further analyses in the present study (Supplementary Figure S1A). In undifferentiated N1E-115 cells, Linx localizes to the tips of short neurites (Fig. 4A). In differentiated N1E-115 cells after serum starvation and in primary cultured hippocampal neurons, Linx also preferentially localizes to the tips of neurites and growing axons (Fig. 4B,C). Furthermore, we also localized Linx to the leading processes of neuroblasts that migrated out from explants isolated from the rostral migratory stream (RMS) of the forebrain (Fig. 4D). These findings imply a role for Linx in the cytoskeletal organization that contributes to the regulation of neurite extension and cell migration.

We next compared morphology as well as neurite extension in control and Linx-depleted differentiated N1E-115 cells, and found that Linx-depleted cells exhibited impaired neurite extension (Fig. 4E). The role of Linx in neurite extension was also demonstrated in primary cultured hippocampal neurons; in these experiments, axonal projections from neurons isolated from the brain of Linx^−/−^ mice were defective compared to those from wild-type brains (Fig. 4F). Intriguingly, hippocampal neurons isolated from Linx^−/−^ brains showed defects in the localization of Tau-1 on growing axons and axon-dendrite differentiation, and an increase in growth cone and cell body areas compared to those from Linx^+/+^ brains. Linx-depleted N1E-115 cells also exhibited an increase in cell body size in both undifferentiated and differentiated cells, which could be rescued by the expression of exogenously added Linx that was fused with green fluorescence protein (GFP; Fig. 4E,G,H). It was noted that the cell body size of hippocampal neurons showed a tendency to be dependent on linx gene dosage, although not statistically proven in the present study (Supplementary Figure S2A). These data suggest a role for Linx in multiple cellular processes that involve neurite elongation and regulation of cell size.

### Linx interacts with Rho-kinase to regulate its activity

**Figure 5:**
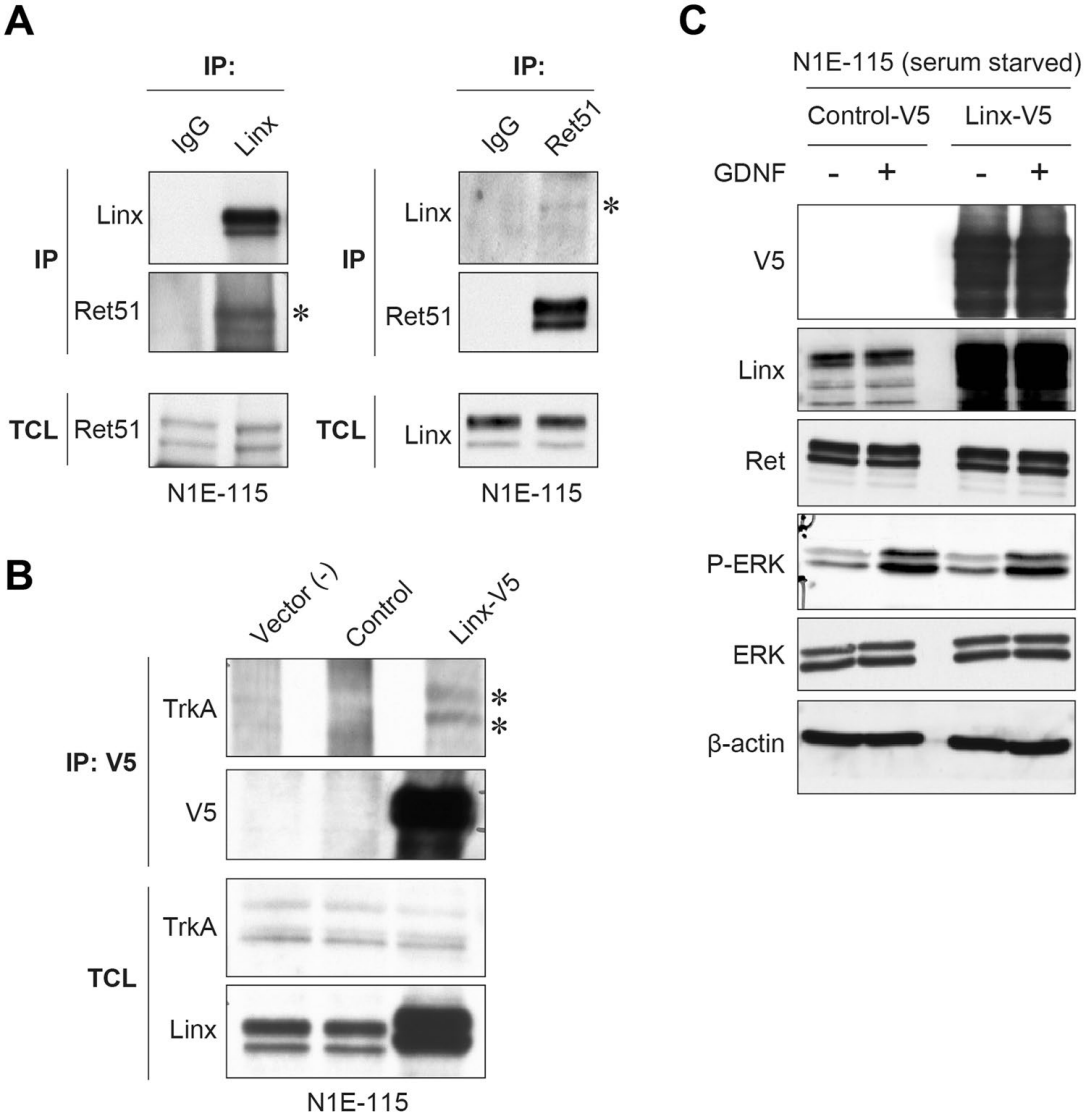
Interaction of Linx with RTKs and its effect on ERK signaling. **(A)** Linx interaction with Ret. Lysates from N1E-115 cells were immunoprecipitated (IP) using Linx (left) and Ret51 (right; an isoform of Ret) antibodies, followed by Western blot analysis with the indicated antibodies. Asterisks indicate co-immunoprecipitated Ret51 and Linx. TCL, total cell lysates. Full blot images are shown in Supplementary Figure S3. **(B)** Linx interaction with TrkA. Lysates from N1E-115 cells transfected with either control or Linx-V5 vector were immunoprecipitated with V5 antibody, followed by Western blot analysis. Asterisks indicate co-immunoprecipitated TrkA. Full blot images are shown in Supplementary Figure S3. **(C)** N1E-115 cells transfected with either control or Linx-V5 vector were starved and stimulated with GDNF, followed by Western blot analysis using the indicated antibodies. Full blot images are shown in Supplementary Figure S3.

A previous study showed the interaction between Linx and RTKs, including Ret, TrkA, and fibroblast growth factor receptor 2 (FGFR2), in which Linx upregulates the signal intensity of growth factor-induced activation of extracellular-regulated kinase (ERK)^4^. In the present study, we confirmed the interaction between Linx and Ret or TrkA in N1E-115 cells using immunoprecipitation (IP) assays (Fig. 5A,B). Unexpectedly, however, we did not observe the effect of Linx overexpression on ERK signaling in N1E-115 cells stimulated with GDNF, the ligand for Ret, suggesting that Linx has other various function(s) that are dependent on context or cell type (Fig. 5C).

**Figure 6:**
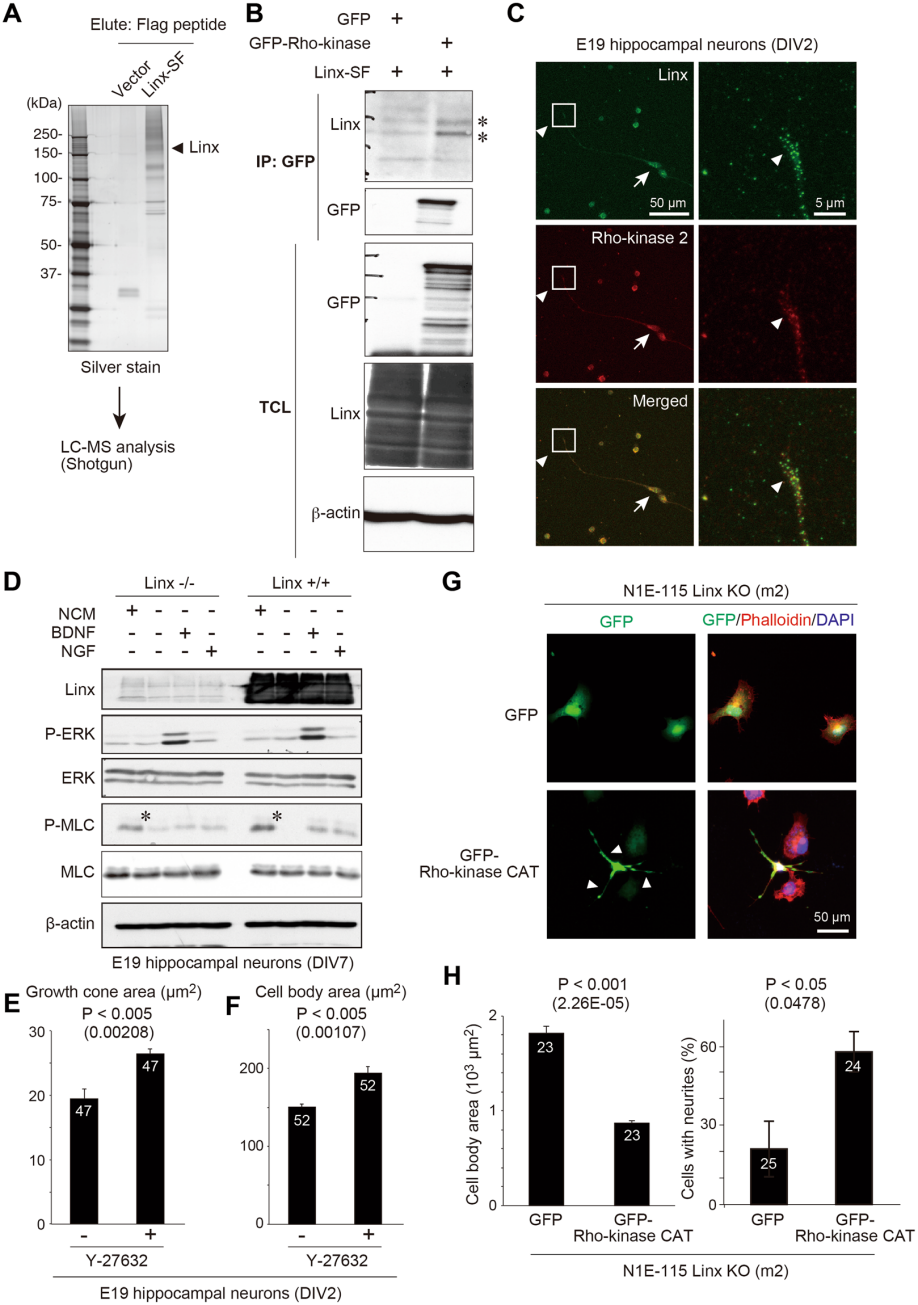
Identification of Rho-kinase as a Linx-interacting protein. **(A)** Representative silver staining of Linx-SF-interacting proteins in 293FT cells isolated by IP. **(B)** 293FT cells were transfected with Linx-SF (streptavidin-Flag) and either GFP or GFP-Rho-kinase, followed by IP using a GFP antibody and Western blot analysis. Asterisks denote co-immunoprecipitated Linx. Full blot images are shown in Supplementary Figure S3. **(C)** Colocalization of endogenous Linx and Rho-kinase at the tips of growth cones (arrowheads) and cell bodies (arrows) of primary cultured hippocampal neurons. Boxed areas were magnified in adjacent panels. **(D)** Linx regulates the activity of Rho-kinase. Hippocampal neurons isolated from the brain of Linx^−/−^ (left) and Linx^+/+^ (right) mice at E19 were incubated in a conventional neuron culture medium (NCM) or starved followed by stimulation with either BDNF, or NGF. Lysates from the neurons were analyzed by Western blot analysis with the indicated antibodies. Note that MLC was more highly phosphorylated in Linx^+/+^ neurons than Linx^−/−^ neurons when cultured in NCM (asterisks). Full blot images are shown in Supplementary Figure S3. **(E**,**F)** Primary cultured hippocampal neurons were isolated from E19 embryo and cultured in the presence or absence of Y-27632 (10 µM) for 5 days, followed the quantification of growth cone **(E)** and cell body **(F)** areas. The numbers in bars indicate the numbers of samples analyzed. **(G**,**H)** Linx-depleted N1E-115 cells (clone m2) were transfected with GFP or GFP-Rho-kinase CAT, followed by staining for the actin filament by Phalloidin and the nuclei **(G)** and the measurement and quantification of cell body area and the numbers of cells with neurites **(H)**. Arrowheads indicate neurites.

To identify new Linx interacting proteins, we generated 293FT human embryonic kidney cells stably expressing Linx fused with a streptavidin-Flag tag (Linx-SF). We immunopurified a protein complex that included Linx-SF by IP, then performed additional analysis of the immunocomplex using mass spectrometry (Fig. 6A). One of the proteins identified to specifically co-immunoprecipitate with Linx-SF was Rho-kinase, a master regulator of the contractility and reorganization of the actin cytoskeleton^24,25^. The interaction between Linx and Rho-kinase 2 was further confirmed by an IP experiment as well as immunofluorescent analysis of cultured hippocampal neurons (Fig. 6B,C), where the specificity of Rho-kinase 2 antibody was shown by neurons depleted of endogenous Rho-kinase 2 by RNA interference (Supplementary Figure S1C,D). We also observed that the phosphorylation (Ser19) of myosin light chain (MLC), a representative substrate of Rho-kinase capable of monitoring its activity, was elevated in neurons isolated from Linx^+/+^ compared to Linx^−/−^ mice when incubated in conventional culture medium (Fig. 6D)^24,26^. The activation of ERK was comparable between neurons from Linx^+/+^ and Linx^−/−^ mice even after stimulation with brain-derived neurotrophic factor (BDNF), the ligand for TrkB (Fig. 6D). The comparison of Linx^+/−^ and Linx^+/+^ neurons, however, resulted in no obvious difference in the phosphorylation of both MLC and ERK, making it unclear to what extent and how Rho-kinase activity is *linx* gene dose-dependently regulated (Supplementary Figure S2B). Furthermore, consistent with the role of Linx in the regulation of cell body size, the inhibition of Rho-kinase activity by its inhibitor Y-27632 resulted in an increase in cell size including growth cone and cell body areas of primary cultured hippocampal neurons (Fig. 6E,F).

Finally, to examine the significance of Linx-mediated regulation of Rho-kinase activity, we transduced GFP (control) or the catalytic domain (CAT) of Rho-kinase, a constitutively active mutant of Rho-kinase, in Linx-depleted N1E-115 cells (clone m2), followed by the measurement of cell body area and neurite extension (Fig. 6G,H). These data, when compared with the data shown in Fig. 4E, showed that the increase in cell body area and the defect in neurite extension in Linx-depleted cells were rescued by Rho-kinase-CAT overexpression (Fig. 6G,H). These data suggest that the Linx/Rho-kinase interaction could be at least relevant to the regulation of cell body size and neurite extension, which may have a role in the development of brain architecture and neuronal connectivity.

## Discussion

In the present study, we found that Linx is a member of the LIG family of membrane proteins that is specifically expressed in neural tissues and essential for the formation of the AC. This study complements a previous study by providing additional evidence for the involvement of Linx in the development of commissural and longitudinal projections in the forebrain^12^. At present, the mechanism underlying the development of hydrocephalus in Linx-deficient and Linx^+/−^ mice remains unclear; however, it could partly be explained by our data demonstrating that Linx is involved in Rho-kinase-mediated regulation of brain cytoarchitecture (Supplementary Figure S2C). The haploinsufficiency of the *linx* gene that was demonstrated in mice, indicates a dose-dependent action of Linx and its physiological significance in brain development.

At present, the mechanism by which Linx functions to regulate neuronal projections remains unclear. A previous study showed that Linx interacts with RTKs including Ret and TrkA, to modulate their intracellular signaling^4^. Although we also identified the biochemical interaction of Linx with Ret and TrkA, we did not find any effects of Linx on RTK-regulated ERK signaling. To our knowledge, the defects in IC and AC formation and the development of hydrocephalus cannot be fully explained by the loss-of-function of either Ret or TrkA, suggesting a role for Linx that is independent of or irrelevant to the functions of those RTKs. This presumption is supported by our recent study that Meflin/Islr, a Linx paralogue that is specifically expressed in mesenchymal stem cells, also interacts with exogenously-expressed RTKs including platelet-derived growth factor receptor, but exhibited no effects on their downstream signaling^27^. Another member of the LIG family of proteins is Lrig1 (leucine-rich repeats and immunoglobulin-like domains 1), which has been well documented to interact with epidermal growth factor receptor (EGFR) and regulate its signaling^8,9,18,19,28–30^. Despite the abundant data reported in the literature, a recent study that revealed the crystal structure of the extracellular domain (ECD) of Lrig1 showed that the purified Lrig1 ECD did not interact with EGFR, questioning the biological significance of the Lrig1/EGFR interaction^31^. Further extended research into potential interacting proteins (ligands) with the members of the LIG protein family will be needed to fully elucidate their function. A biochemical screen to identify proteins that interact with the zebrafish orthologue of Linx/Islr2 has identified Vasorin as a candidate ligand for the Linx ECD^32^. Vasorin, also termed Slit-like 2 (SLITL2), is a known regulator of neural and vascular morphogenesis that is widely expressed among species^33,34^. Future studies should thus focus on identifying if the Linx/Vasorin interaction is also present in the brains of mammals.

Interestingly, Linx depletion had a significant effect on neurite extension in differentiated N1E-115 cells induced by serum starvation, indicating that Linx may function cell-intrinsically or independently of any growth factor(s) stimulation to promote neurite outgrowth. In the present study, by monitoring MLC phosphorylation we found that Linx interacts with and regulates the activity of Rho-kinase, a regulator of actin cytoskeletal organization and remodeling^25^. We believe this is consistent with the effects of Linx depletion on neurite extension in N1E-115 cells and hippocampal neurons. How Linx controls the activity of Rho-kinase and the Linx/Rho-kinase complex is involved in the development of brain architecture including the AC, however, remain to be determined and will be the subject of future studies (Supplementary Figure S2C). Our preliminary experiments showed that activity of the small GTPase RhoA was also upregulated by Linx overexpression in N1E-115 cells (data not shown); however, this needs to be confirmed by more extensive biochemical investigations.

Another intriguing issue surrounding Linx function is the complexity by which Linx controls axonal projections, which appears to be more complicated than Linx simply regulating cytoskeletal organization. A previous study showed that Linx is not only expressed in corticofugal axons descending through the IC, but is also expressed along their route which is comprised of the prethalamus and lateral ganglionic eminence (LGE)-derived corridor^12^. Moreover, Linx binds to thalamocortical projections ascending through the IC, which helps the reciprocal intermingling of axons from multiple neurons^12^. These data imply that Linx is not a simple guidance cue molecule but is also involved in cell-cell interactions or intercellular communication via unknown mechanisms. Detailed examination of the Linx expression pattern at higher resolution will be required to further address this issue.

## Methods

### Ethical approval

All animal protocols were approved by the Animal Care and Use Committee of Nagoya University Graduate School of Medicine. All *in vivo* experiments were performed in compliance with Nagoya University’s Animal Facility regulations.

### Cell culture

N1E-115 and NIH3T3 cell lines were purchased from American Type Culture Collection (ATCC). The 293FT cell line was purchased from Clontech. The sources of other human cell lines used in the study were previously described^35–38^. All cell lines were cultured in Dulbecco’s modified Eagle’s medium (DMEM) containing 10% fetal bovine serum (FBS). For the isolation of hippocampal neurons and their primary culture in neuron dissociation solutions and neuron culture medium (Wako, Japan), we followed a previously described procedure^39^.

### Plasmids and Antibodies

cDNA encoding mouse Linx (clone 6826287, GenBank accession number BC096531) was purchased from Open Biosystems and subcloned into pcDNA3.1 (Invitrogen) and pRetroQ (Clontech) vectors to fuse Linx with the V5 and SF tag, respectively. GFP-Rho-kinase cDNA was provided by M. Amano and K. Kaibuchi (Nagoya University). The following antibodies were used in this study; anti-Linx (Islr2; R&D Systems), anti-Linx (Islr2; Abnova), anti-Ret51 (IBL, Gumma, Japan), β-actin (Sigma), anti-Tau-1 (Millipore), anti-TrkA (Cell Signaling Technology), anti-phospho-MLC (Ser19; Cell Signaling Technology), anti-MLC (Cell Signaling Technology), anti-Rho-kinase 2 (ROCK2, Abcam), anti-L1 (Millipore), anti-E-cadherin (Cell Signaling Technology), anti-Na^+^/K^+^-ATPase (Abcam), and anti-GFP (MBL, Nagoya, Japan).

### Western blot analysis and IP

We followed a standard protocol for Western blot analysis as described previously^27^. For IP, cultured cells were lysed on ice in a chilled buffer containing 20 mM Tris-HCl (pH 7.4), 120 mM NaCl, 1 mM EDTA, and 1% Triton X-100 supplemented with Complete protease inhibitor cocktail (Roche) and PhosSTOP phosphatase inhibitor cocktail (Roche). The lysates were centrifuged at 12,000 × g for 10 min prior to conventional IP using the indicated antibodies and Western blot analyses. For Western blot analysis, the primary antibodies were diluted with Can-Get-Signal Solution 1 (Toyobo, Osaka, Japan) to enhance antibody-antigen binding.

### Linx-deficient mice

Targeted embryonic stem (ES) cell clone (KO-1319, Islr2 AD7) isolated from VGB6 cells (C57BL/6NTac background) were purchased from the KOMP Repository (www.komp.org). Quality control for correct targeting and homologous recombination in the 5′ and 3′ homology arms was assessed by long range PCR by the KOMP. The *linx/islr2* gene consists of three exons, where exon 3 contained the entire open reading frame (ORF; Fig. 2A). The targeting construct was designed by the KOMP to replace the whole Linx ORF (exon 3) with the LacZ-loxP-neo-loxP cassette and later delete the neo-resistance gene via Cre recombinase (Fig. 2A). The targeted ES cells were injected into Balb/c blastocysts by the Institute of Immunology Co. Ltd. (Tokyo, Japan) and the resulting chimeric male mice were mated with C57BL/6 J female mice to generate F1 animals heterozygous for the cassette, which were then crossed with actin-Cre transgenic mice to remove the neo-resistance gene.

### Immunofluorescence staining

N1E-115 cells and hippocampal neurons isolated from the brains of embryonic day 19 (E19) wild-type and Linx^−/−^ mice were grown on poly-D-lysine (PDL)-coated glass bottom dishes (Iwaki, Japan), fixed in 4% (w/v) paraformaldehyde (PFA), permeabilized with phosphate-buffered saline (PBS) containing 0.05% (v/v) TritonX-100 and then incubated with the indicated primary antibodies, followed by staining with Alexa 488/594-conjugated goat anti-mouse or anti-rabbit IgG (Invitrogen). Nuclei were visualized by DAPI (4′6-diamidino-2-phenylindole) staining. After washing in PBS, fluorescence was visualized with a confocal laser scanning microscope (LSM 700, Carl Zeiss) or a fluorescent microscope BZ-X700 (Keyence, Japan).

### X-gal staining of brain slices

X-gal staining of brain slices was performed as described previously^40^. Briefly, brains of wild-type and Linx^+/−^ mice were perfused with 4% paraformaldehyde in 0.1 M phosphate buffer and manually cut into 1 mm-thick sections. The sections were then fixed in LacZ fixative (0.1% glutaraldehyde and 2 mM MgCl_2_ in PBS) for 10 min on ice, washed twice in LacZ washing buffer (2 mM MgCl_2_, 0.01% sodiumdeoxycholate, and 1% Nonidet P-40 in PBS) for 10 min, and stained with X-Gal staining solution (1 mg/ml X-Gal, 5 mM potassiumferrocyanide, and 5 mM potassiumferricyanide in LacZ washing buffer) for 1 to 3 days at 37 °C. After color development, sections were washed in PBS and mounted. Images were acquired using a light microscope (BX-50, Olympus).

### Fluorescent labelling of AON neurons

Whole brains of P7 wild-type and Linx^+/−^ mice were perfused and fixed with 4% paraformaldehyde in 0.1 M phosphate buffer. A small crystal of DiI was placed onto the AON region using fine tweezers, followed by incubation at 37 °C for 3 days; the brains were then optically cleared using the SeeDB (See Deep Brain) method. Images were acquired using a light microscope (BX-50, Olympus).

### Generation of Linx knockout cells using the CRISPR/Cas9 genome editing

The 20 nucleotide guide sequences targeting the mouse *linx* gene were designed using the CRISPR design tool (http://www.genome-engineering.org/crispr/) and cloned into a lentivirus expression vector lentiCRISPR v2 (Addgene #52961)^23^. The guide sequences targeting the mouse *linx* gene were 5′-GCGCACCCCCCAGCGTGCGT-3′ (m2) and 5′-ATGTTACATTGCGTCGCCGA-3′ (m3). The single guide RNAs (sgRNAs) in the lentiCRISPR v2 vector (2.5 μg), pxPAX2 (2.5 μg) and pVSV-G (1 μg) were transfected into 293 T cells in 6-cm dishes using Lipofectamine 2000 (Invitrogen) according to the manufacturer’s instructions. Twenty-four hours post-transfection, the medium was replaced with 5 ml of fresh DMEM/10% FBS. Seventy-two hours post-transfection, the medium was collected and syringe filtered with a 0.45 µm pore size membrane (Millipore). The collected medium containing the virus was used to infect N1E-115 cells, followed by incubation for 48 hr with the addition of puromycin (1 µg/ml) for selection.

### Mass spectrometry

Total cell lysates from control 293FT cells and those stably expressing mouse Linx-SF (streptavidin-Flag) were immunoprecipitated with an anti-Flag antibody (Sigma), followed by elution of the protein complex including mouse Linx-SF with the Flag peptide (Sigma). The eluate was separated by sodium dodecyl-polyacrylamide gel electrophoresis (SDS-PAGE) and detected by silver staining (SilverQuest Silver Staining Kit, Invitrogen) following the manufacturer’s instructions. For a shotgun approach to identify proteins in the eluate, whole eluates were digested with Trypsin Gold (Promega) for 16 h at 37 °C after reduction, alkylation, demineralization, and concentration, followed by analysis on an Orbitrap Fusion mass spectrometer (Thermo Fisher Scientific).

### RNA interference

For RNA interference-mediated knock down of endogenous Rho-kinase 2, we purchased the pool of four different small interfering RNAs (siRNAs) that are predesigned by Dharmacon. We transfected control siRNA (Dharmacon) and the Rho-kinase 2 siRNA into N1E-115 cells using Lipofectamine 2000 (Invitrogen).

### Data analysis

Data are presented as the mean ± S.D. Statistical significance was evaluated with Student’s *t* test.

### Data availability

The datasets generated and/or analyzed during the current study are available from the corresponding author on reasonable request.

## Electronic supplementary material

Supplementary Information
