# Supplementary material for: Essential Role of Linx/Islr2 in the Development of the Forebrain Anterior Commissure
Source: Sci Rep. 2018 May 8;8:7292. doi: 10.1038/s41598-018-24064-0 (PMC5940738; doi:10.1038/s41598-018-24064-0)
Supplement: Supplementary file 1 — Supplementary Information [file 41598_2018_24064_MOESM1_ESM.pdf]

Essential Role of Linx/Islr2 in the Development of the Forebrain Anterior Commissure

Shaniya Abudureyimu, Naoya Asai, Atsushi Enomoto, Liang Weng, Hiroki Kobayashi, Xiaoze Wang, Chen Chen, Shinji Mii, Masahide Takahashi

Supplementary Information

- Figure legends for Supplementary Figure S1-2
- Supplementary Figure S1-2
- Supplementary Figure S3: Unprocessed blot images

Supplementary Fig. S1. Specificity of antibodies used in the study.

(A) CRISPR/Cas9-based depletion of endogenous Linx in N1E-115 cells. Full blot images are shown in Supplementary Figure S3.

(B) Control and Linx-depleted (Linx KO) N1E-115 cells were plated on glass base dishes, followed by staining with anti-Linx antibody (R&D Systems). The antibody detected signals on the control cells (upper panel), which were attenuated in Linx KO cells. bars, 50 μm.

(C) HeLa cells were transfected with control and Rho-kinase 2 siRNAs and incubated for 48 hours, followed by Western blot analysis using Rho-kinase 2 and β-actin antibodies.

(D) Hippocampal neurons cotransfected with GFP and the indicated siRNAs were stained with Rho-kinase 2 antibody. Note that the staining of neurites with Rho-kinase 2 antibody (arrowheads) was attenuated in Rho-kinase 2 siRNA-transfected cells.

Supplementary Fig. S2. Linx-mediated regulation of cell body size and Rho-kinase activity requires full expression of both *linx* alleles.

(A) Hippocampal neurons isolated from the brains of Linx$^{+/+}$, Linx$^{-/-}$, and Linx$^{+/-}$ embryos at E19 were cultured for 2 days, followed by the measurement of cell body

area. The numbers in bars indicate the numbers of samples analyzed. N.S., not significant.

(B) Hippocampal neurons isolated from the brains of Linx$^{+/+}$ (left) and Linx$^{+/-}$ (right) mice at E19 were incubated in a conventional neuron culture medium (NCM) or starved, followed by stimulation with BDNF. Lysates from the neurons were analyzed by Western blot analysis using the indicated antibodies. Note that Myosin light chain (MLC) phosphorylation was comparable between Linx$^{+/+}$ and Linx$^{+/-}$ neurons when they were cultured in NCM (asterisks). There also seemed to be no difference in the activation of ERK between Linx$^{+/+}$ and Linx$^{;/-}$ neurons when they were stimulated with BDNF.

(C) A working hypothesis and limitations of the present study. The present study showed that defective expression of Linx is responsible for the development of hydrocephalus and defective anterior commissure (AC). Linx$^{+/-}$ mice also develop these phenotypes, suggesting haploinsufficiency of the *linx* gene in mice. Our cell biological analysis showed the possibility that Linx may regulate cell body size and neurite extension through the regulation of Rho-kinase activity, which, however, has not been fully proven in the present study. The relevance of Linx-mediated regulation of cell size and neurite extension in the development of hydrocephalus and defective AC also remains unknown at present.

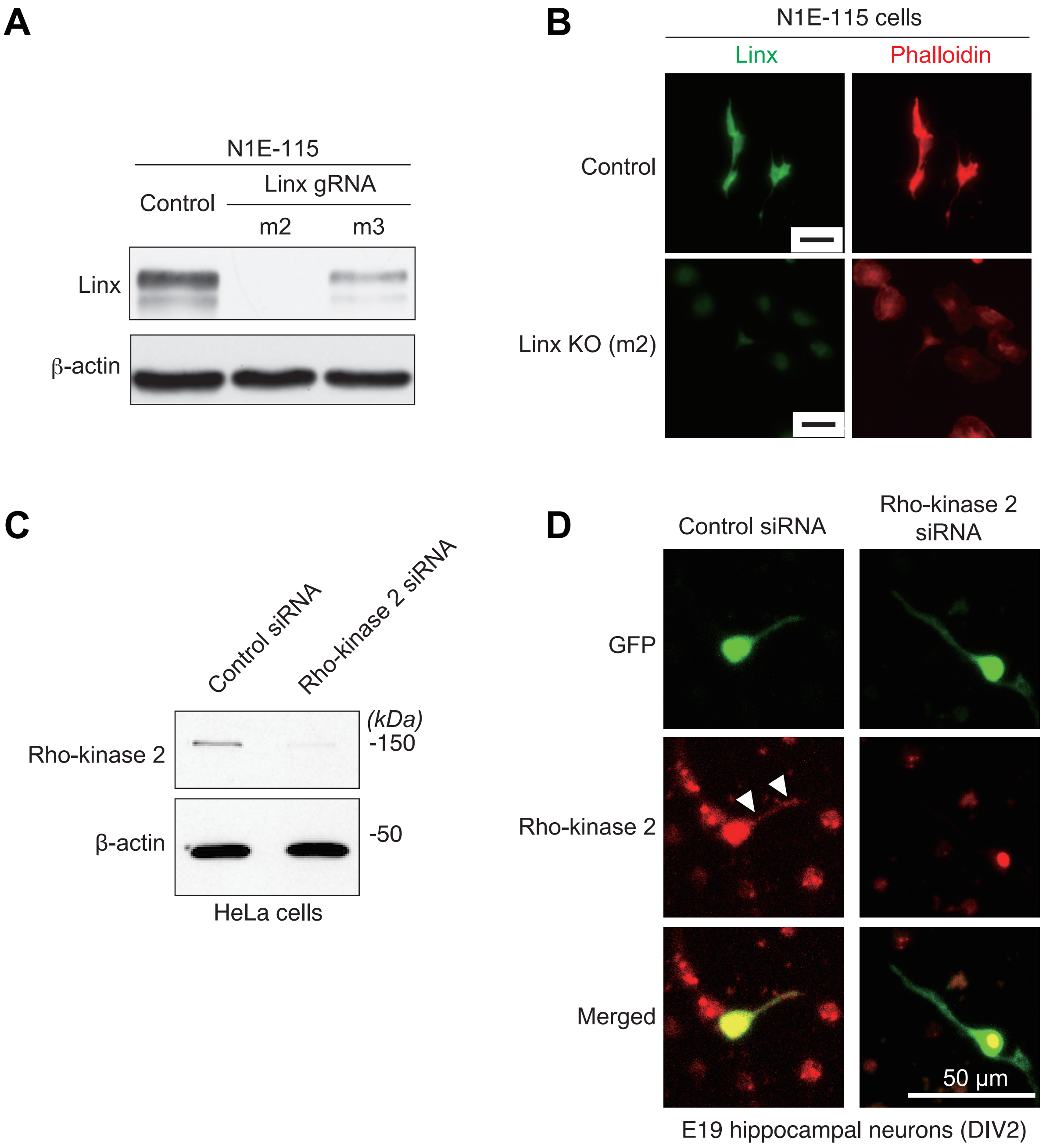

Abudureyimu et al., Supplementary Figure S1

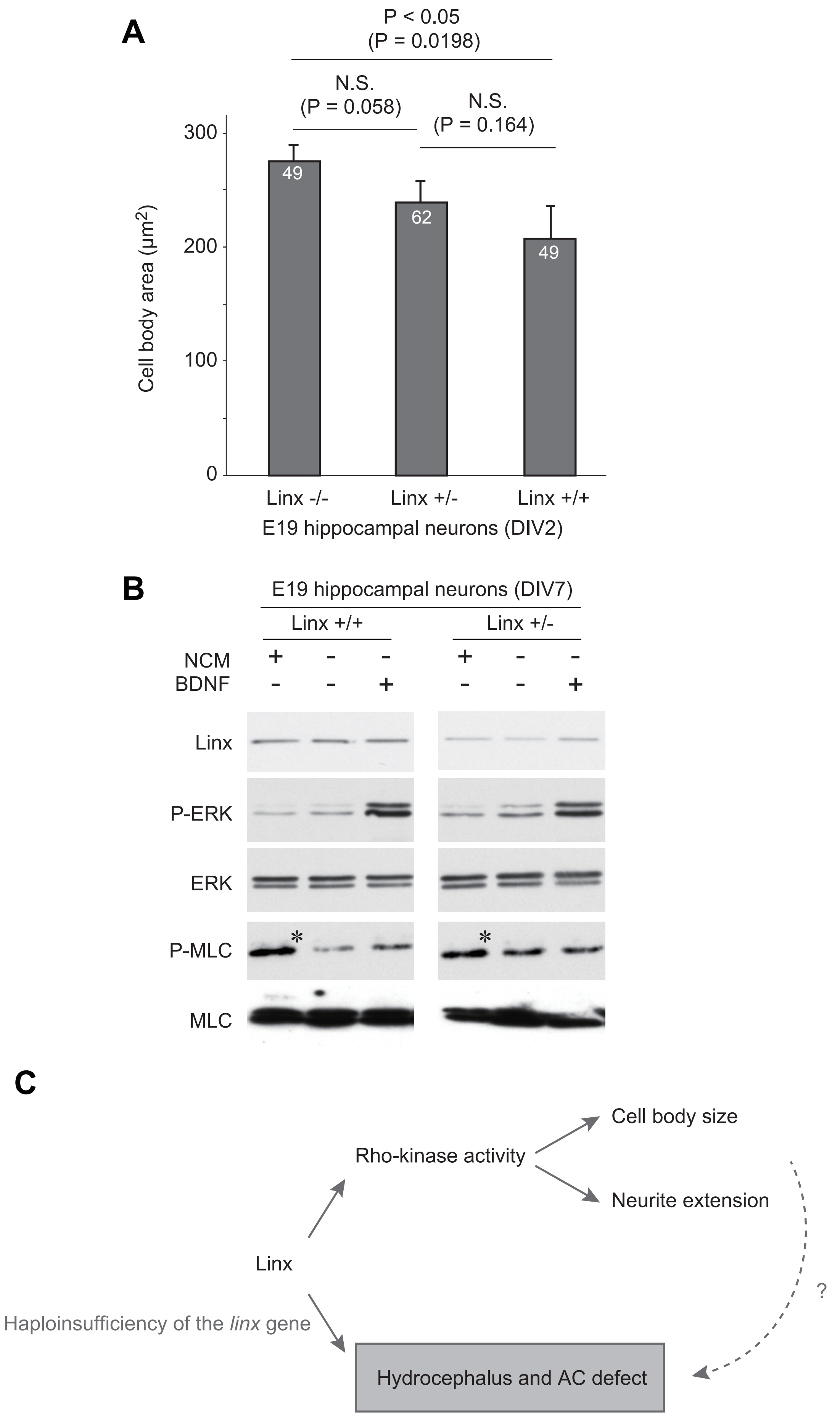

Abudureyimu et al., Supplementary Figure S2

Unprocessed blot images

Figure 1B

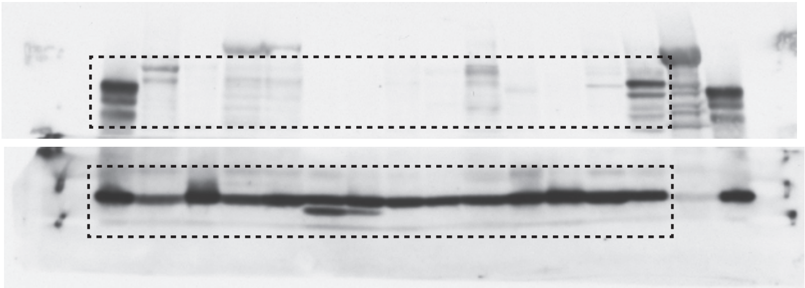

Figure 1C

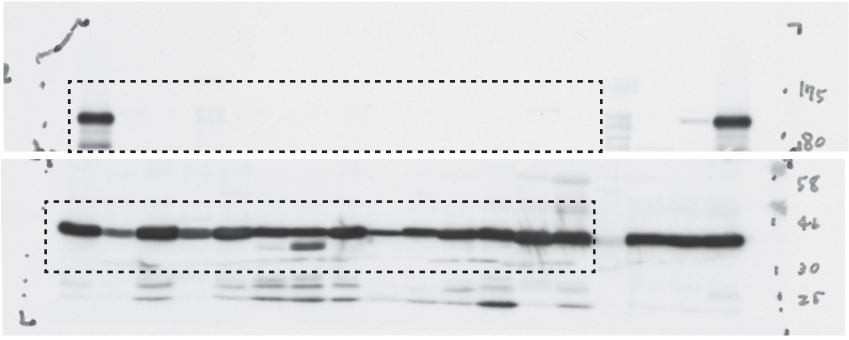

Figure 1D

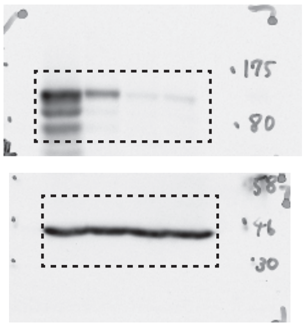

Figure 1E

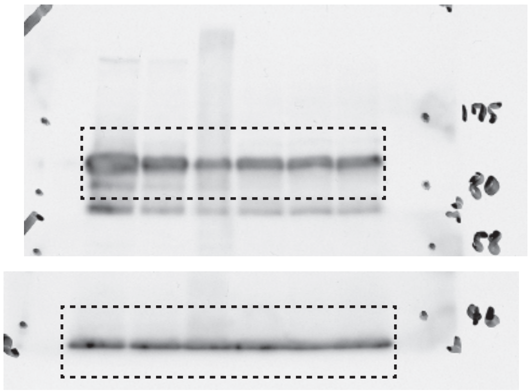

Figure 1F

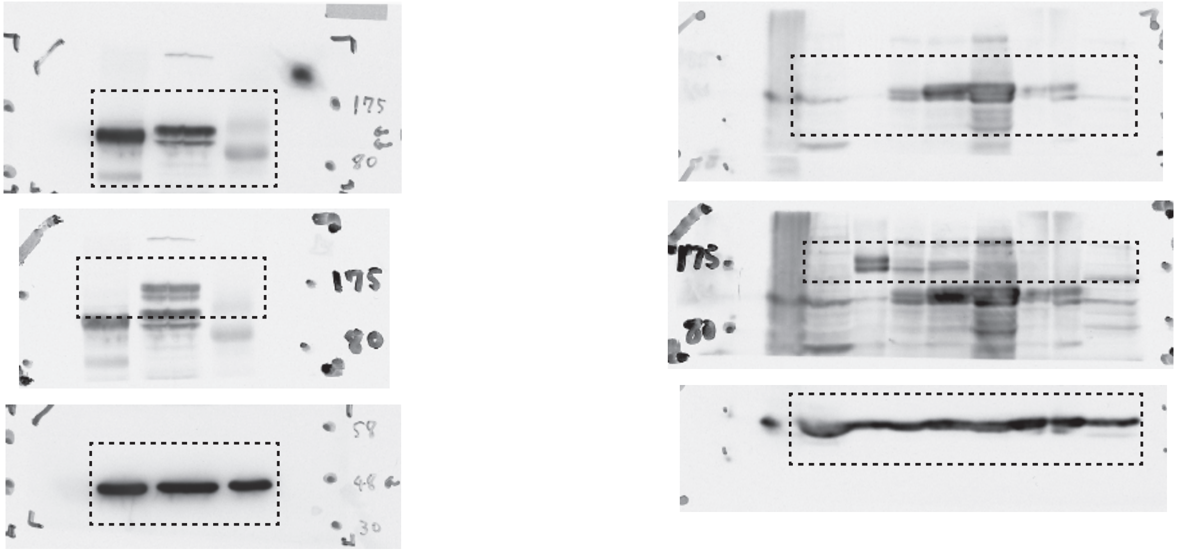

Abudureyimu et al., Supplementary Figure S3

Unprocessed blot images

Figure 4G

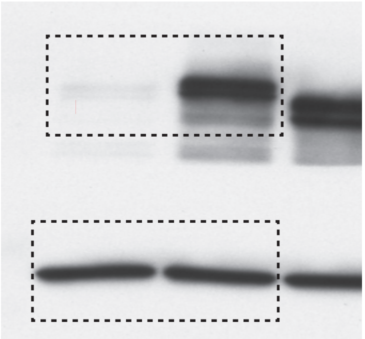

Figure 5A

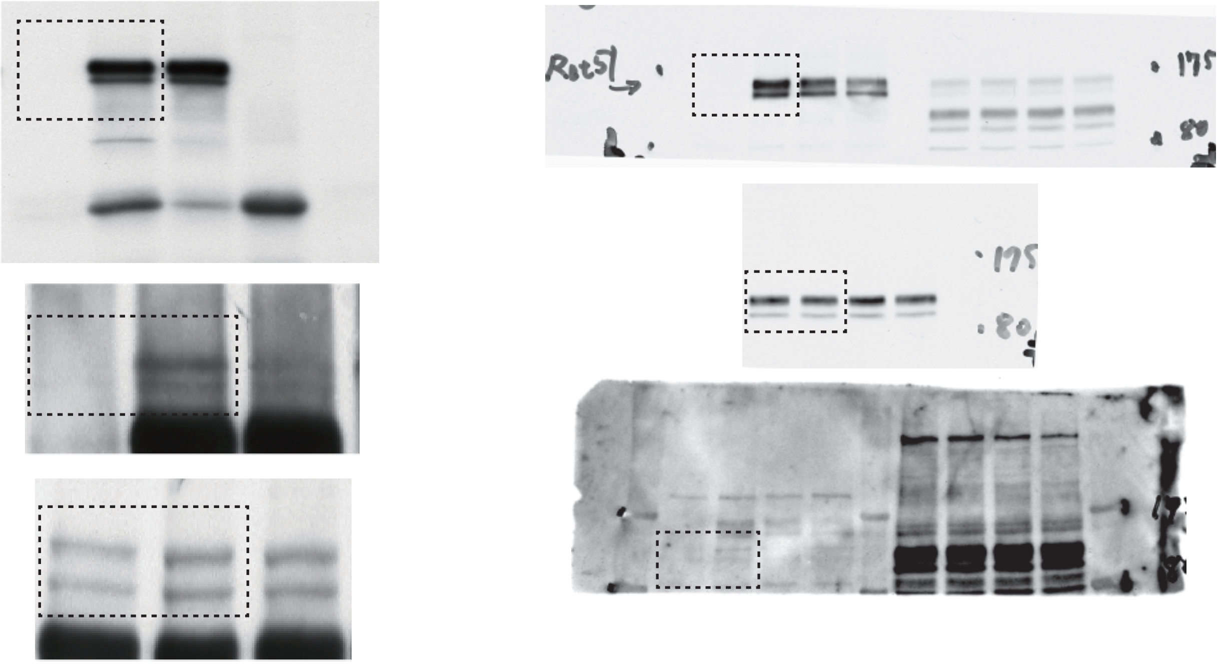

Abudureyimu et al., Supplementary Figure S3

(Continued)

Unprocessed blot images

Figure 5B

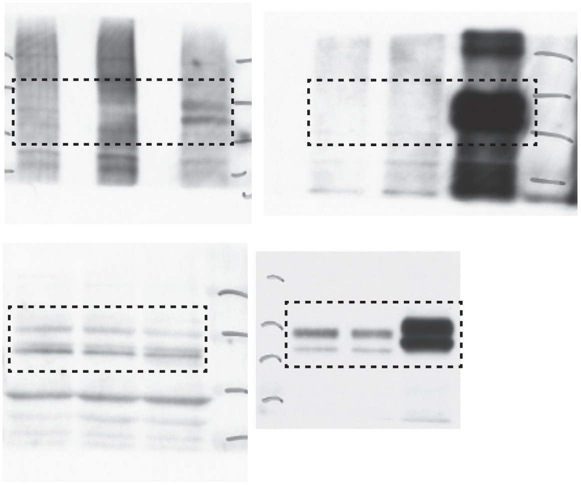

Figure 5C

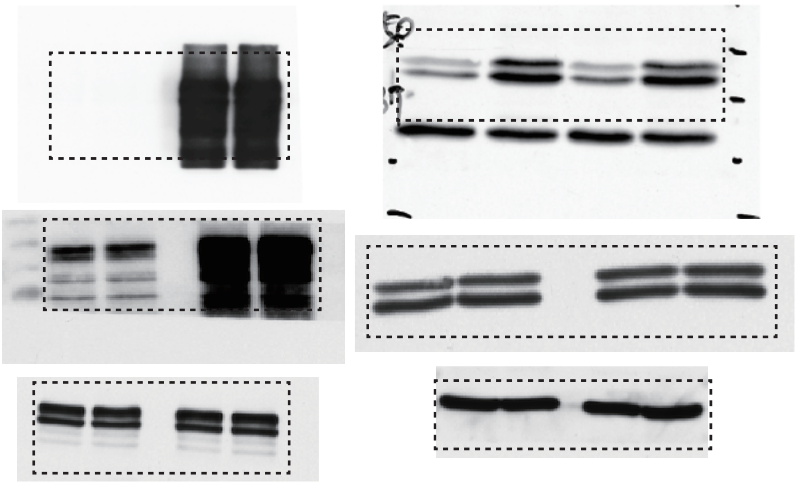

Figure 6B

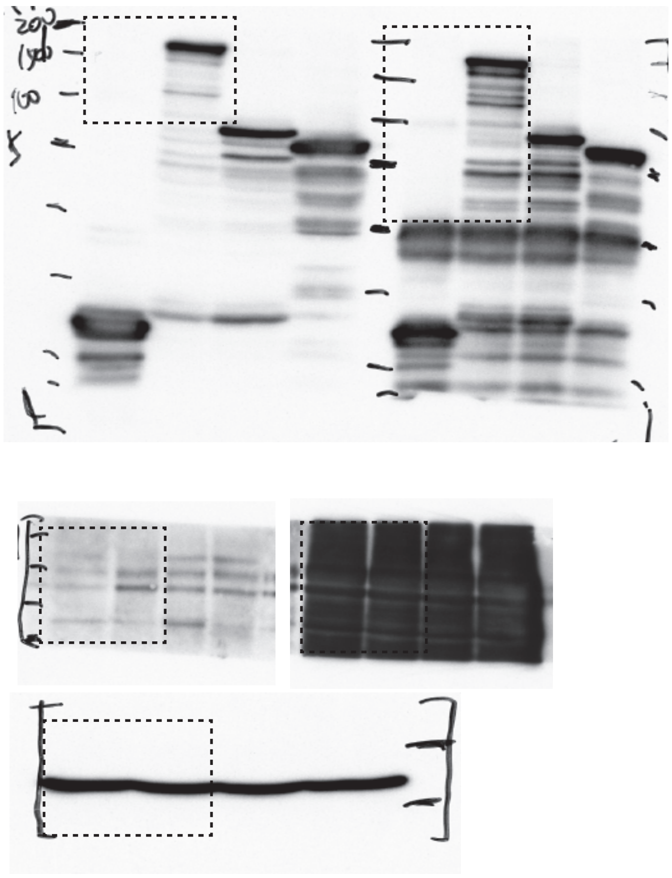

Figure 6D

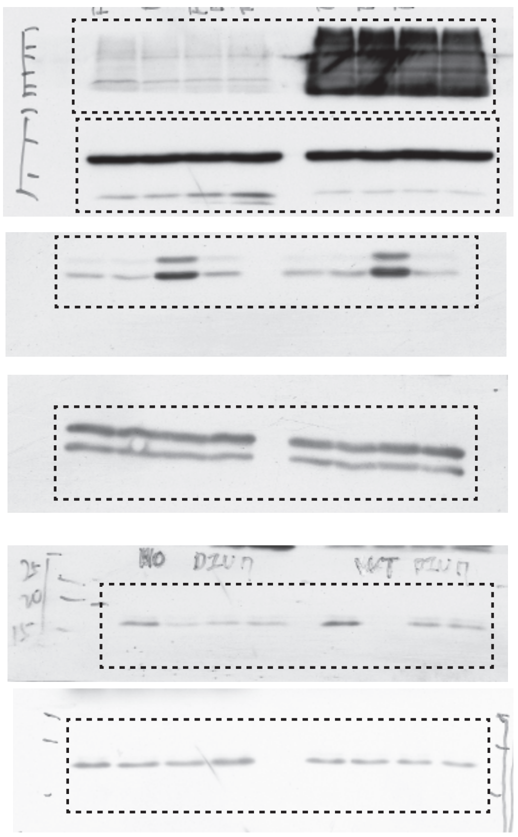

Abudureyimu et al., Supplementary Figure S3

(Continued)

Unprocessed blot images

Supplementary Fig. S1A

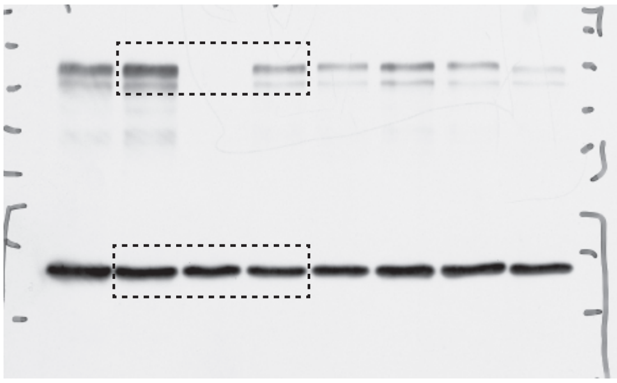

Supplementary Figure S1B

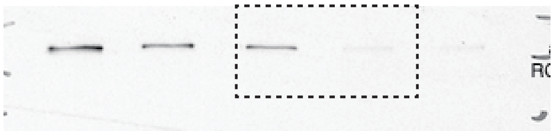

Supplementary Figure S2B

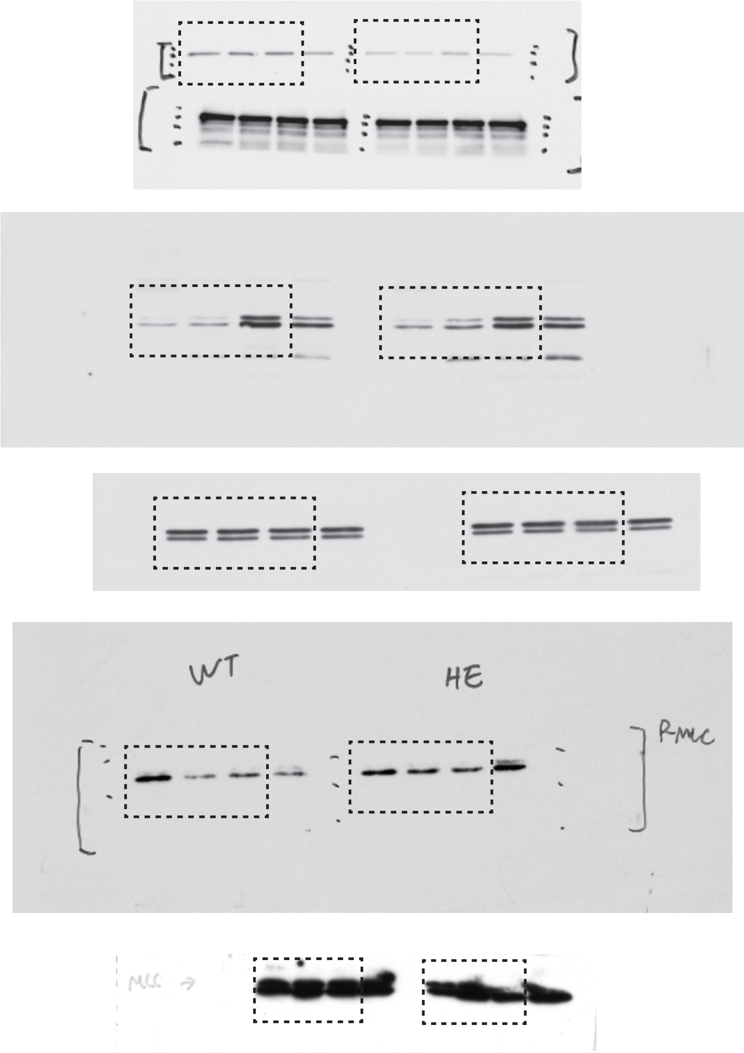

Abudureyimu et al., Supplementary Figure S4

(Continued)
